# Suggestive Therapeutics

**Published:** 1889-07

**Authors:** 


SUGGESTIVE THERAPEUTICS, a treatise on the nature and uses of hypnotism, by H. Bernheim, M. D., Prof. in the Faculty of Medicine at Nancy. Translated from the second and revised French edition, by Christian A. Herter, M. D. of New York. New York and London. G. P. Putnam's Sons, 1889.

We have here a very interesting work of 416 oc. pages, giving useful and practical information upon a very important subject. Hypnotism or Mesmarism is now a conceded fact. It matters not whether the phenomena are caused by an occult force in nature, or whether they are due to the imagination, they are nevertheless true and remarkable. It is evident also, that, in whatever it consists, it is a power for good or for evil. As a therapeutic agent it is clearly capable of accomplishing great good, and in the hand of the honorable and consciencious physician should be used with caution and great discretion.

The present work is full upon the methods to be used, and gives numerous cases and facts illustrative of its uses.

